# Successful Use of Octreotide Therapy for Refractory Levofloxacin-Induced Hypoglycemia: A Case Report and Literature Review

**DOI:** 10.1155/2019/3560608

**Published:** 2019-05-09

**Authors:** Matthew R. Watson, Ceressa T. Ward, Amit Prabhakar, Babar Fiza, Vanessa Moll

**Affiliations:** ^1^Pharmaceutical Services, Emory University Hospital Midtown, Atlanta, Georgia, USA; ^2^Department of Anesthesiology, Emory Center of Critical Care, Emory School of Medicine, Atlanta, Georgia, USA

## Abstract

Fluoroquinolones are commonly prescribed antimicrobials that have been implicated in alterations of glucose metabolism. We report a case of refractory fluoroquinolone-induced hypoglycemia in a patient with type-2 diabetes mellitus on glipizide that was successfully treated with octreotide. A patient was admitted with hypoglycemia after having been initiated on levofloxacin therapy. Despite treating the hypoglycemia supportively with multiple boluses of 25 g of dextrose, a continuous dextrose infusion, and glucagon, the patient experienced repeated episodes of rebound hypoglycemia. The persistent hypoglycemia was eventually reversed with the administration of subcutaneous octreotide. Clinicians should be cognizant of this adverse effect of fluoroquinolones, as well as predisposing risk factors, and consider octreotide as an adjunctive therapy for refractory hypoglycemia cases.

## 1. Introduction

Fluoroquinolones are a commonly prescribed class of broad-spectrum antimicrobials used for a variety of bacterial infections given their excellent degree of tissue penetration and high oral bioavailability [1]. Although appropriate for select indications, routine use of fluoroquinolones has been questioned due to associated risks. Serious adverse effects have been linked to fluoroquinolones prompting recent updates to the safety labeling which now includes potential risk for significant hypoglycemia resulting in coma [2]. The mechanism of fluoroquinolone-induced hypoglycemia is poorly understood. However, it is postulated that fluoroquinolones interact with insulin producing pancreatic *β*-cells. Currently, there are no targeted therapeutic options for treating this adverse effect. Given the hypothesized mechanism, octreotide may represent a novel treatment for reversal of fluoroquinolone-induced hypoglycemia. We report a case of severe life-threatening and refractory hypoglycemia from levofloxacin successfully treated with octreotide.

## 2. Case Report

A 73-year-old Caucasian male with a past medical history of coronary artery disease, heart failure, atrial fibrillation, chronic obstructive pulmonary disease, and type-2 diabetes mellitus was admitted after having a witnessed seizure at his nursing rehabilitation facility. When emergency medical services arrived, the patient was found to be hypoglycemic with blood glucose (BG) of 21 mg/dL. He was administered 25 g of dextrose 50% (D50) resulting in some improvement in his mental status. He was then transferred to our emergency department (ED).

In the ED, the patient was minimally responsive to both verbal and physical cues. The initial laboratory results were significant for hypokalemia with potassium of 2.9 mmol/L (normal 3.6-5.1 mmol/L), acute kidney injury with serum creatinine at 2.52 mg/dL (normal 0.7-1.3 mg/dL; baseline approximately 1.5 mg/dL), albumin of 2 g/dL (normal 3.5-5.7 g/dL), and hypoglycemia with a BG of 34 mg/dL (normal 70-105 mg/dL). Liver function tests (LFTs) were all within normal limits. The patient was immediately given 50 g of D50 resulting in a repeat BG of 134 mg/dL. Review of the patient's home medication list identified that the patient was prescribed levofloxacin 750 mg daily for pneumonia on discharge from a hospitalization three days prior with no other medication changes. Chronic medications were inclusive of aspirin 81 mg daily, atorvastatin 80 mg daily, glipizide 10 mg daily, losartan 25 mg daily, mometasone 220 mcg/inhalation three times daily, spironolactone 25 mg daily, torsemide 100 mg daily, and warfarin. The patient required four additional boluses of D50 and a continuous dextrose 10% (D10) infusion to maintain euglycemia before transfer to the intensive care unit (ICU).

**Figure 1:**
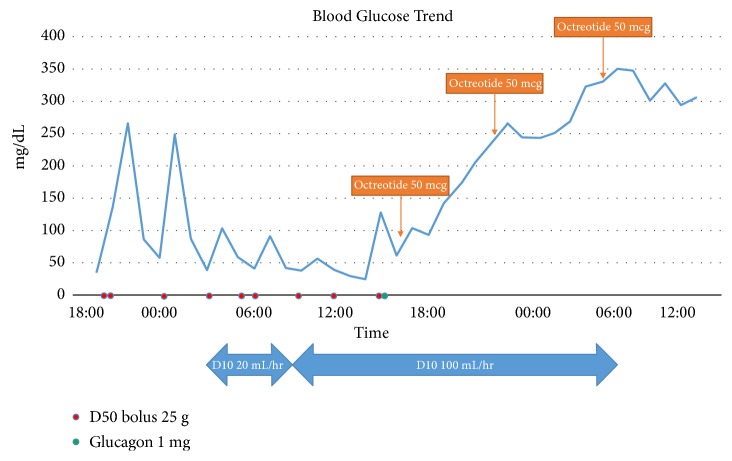
Blood glucose values in response to D10 infusion, D50 boluses, glucagon, and octreotide. D10, dextrose 10%; D50, dextrose 50%.

In the ICU, the patient continued to experience multiple episodes of severe rebound hypoglycemia despite an additional four boluses of D50, increasing the D10 infusion rate to 100 mL/hr (see Figure 1) and withholding home medications. Glucagon 1 mg intramuscularly only led to a transient increase in BG. Given the patient's refractory hypoglycemia to standard supportive care therapies, octreotide 50 mcg subcutaneously was administered every six hours for a total of three doses. Within three hours of administration of the first dose of octreotide, there was a significant increase in BG levels (ranging from 170 to 237 mg/dL) and no further D50 boluses were required. Due to persistent hyperglycemia (BG >250 mg/dL), the D10 infusion was discontinued 5.5 hours after the second dose of octreotide. The patient eventually required an insulin infusion the following day and was transferred to the floor. The patient was ultimately discharged to his nursing rehabilitation facility in stable condition.

## 3. Discussion

Hypoglycemia is a rare but a known potential adverse effect of fluoroquinolone therapy. Several published case reports have specifically implicated levofloxacin as the causative agent of hypoglycemia with some resulting in fatal outcomes [3–13]. This case report adds to the emerging data documenting fluoroquinolone-induced hypoglycemia. The exact incidence of this adverse effect is not known. However, a total of 67 cases of fluoroquinolone-associated hypoglycemia have been identified through review of the FDA Adverse Event Reporting System (FAERS) and published literature from 1987 to 2017 [14]. This figure is likely conservative due to probable underrecognition and thus underreporting. Due to hypoglycemia having the potential to lead to serious morbidity and mortality, it is important for clinicians to recognize risk factors associated with this adverse event and increase monitoring or choose alternative therapy when appropriate [15].

Several risk factors may predispose patients to hypoglycemia while being treated with fluoroquinolones including diabetes, concomitant use of sulfonylureas or insulin, renal insufficiency, and elderly which is conventionally defined as greater than 65 years old [2, 16, 17]. Although levofloxacin was dosed appropriately for renal function at the time of prescription for pneumonia, our patient did have multiple risk factors (i.e., elderly with diabetes on glipizide) that could have prompted discussion regarding alternative treatment. If a fluoroquinolone is determined to be the most optimal antibiotic based on indication, there is data to support that differences in risk of hypoglycemia exist among this class that could assist in guiding therapy.

In a large population-based cohort study of over 78,000 diabetic patients newly prescribed a fluoroquinolone, the absolute risk of hypoglycemia per 1,000 people for moxifloxacin, levofloxacin, and ciprofloxacin was found to be 10.0, 9.3, and 7.9, respectively. This is in comparison to 3.7 for macrolides and 3.2 for cephalosporins [18]. In contrast to this study, the FAERS found moxifloxacin to be the least associated with hypoglycemia. Moxifloxacin, ciprofloxacin, and levofloxacin accounted for 9, 12, and 44 of the 67 cases of hypoglycemia reported, respectively. This data is consistent with published work from the Veteran Affairs system where levofloxacin was noted to confer a higher risk of hypoglycemia in comparison to ciprofloxacin. Moxifloxacin was not studied in that publication due to low usage at the time of the study [19]. The interpretation of this data may be limited by reporting/publication bias. Nonetheless, the differences can likely be explained by variations in molecular structure which have been seen with other toxicities associated with fluoroquinolones [20].

The mechanisms by which fluoroquinolones induce hypoglycemia have not yet been fully elucidated. However, in vitro and animal model studies have provided evidence on proposed pharmacodynamic pathways that modulate insulin secretion. These studies in addition to the known pharmacokinetic profile of levofloxacin provide potential explanations for this clinical scenario.

Adenosine triphosphate-sensitive potassium (KATP) channels located in pancreatic *β*-cells play a key role in sensing plasma glucose levels and triggering the release of insulin to maintain euglycemia [21, 22]. In vitro studies suggest that fluoroquinolones block this channel resulting in membrane depolarization leading to an influx of calcium through voltage-gated calcium channels and subsequent increase in insulin release [23, 24]. These similar molecular mechanisms occur when sulfonylureas bind to the sulfonylurea receptor 1 subunit on the KATP channels. This results in the same downstream signaling resulting in exocytosis of insulin secretory granules via calcium signaling [25]. Due to the varying risks of hypoglycemia reported in the literature among the fluoroquinolones, further research is needed to understand possible unidentified biochemical triggering factors [26].

In addition to pharmacodynamics, a pharmacokinetic mechanism in our case should be explored. On admission, our patient was noted to be on concomitant therapy with glipizide for type-2 diabetes. Hence, the possibility of a combined drug-drug interaction between levofloxacin and glipizide should be considered as another mechanism to explain the refractory hypoglycemia. Glipizide is primarily metabolized by the cytochrome P-450 (CYP) system, specifically isoenzyme CYP2C9 [27]. It has been shown in an in vitro model of human liver microsomes that levofloxacin weakly inhibits CYP2C9 [28]. Due to the existence of genetic polymorphisms of CYP2C9, patients may exhibit different baseline metabolism rates that variably change by the addition of a CYP2C9 inhibitor. This could possibly explain the differences in clinical outcomes and/or adverse events between patients on these two medications. Moreover, levofloxacin and glipizide are approximately 24-38% and 98-99% protein bound primarily to albumin, respectively [29, 30]. Given these ratios, albumin binding affinities, and different primary albumin binding sites, the probability of levofloxacin causing a significant displacement of glipizide to increase its free drug fraction, thus its therapeutic/adverse effects, is unlikely [31, 32].

Although supportive care therapies such as glucose and glucagon administration remain the cornerstone of treatment for hypoglycemia, complications have been reported. In this therapeutic approach, transient beneficial increases in serum glucose are offset by rebound hypoglycemia that can especially occur in patients taking medications affecting pancreatic KATP *β*-cell channels such as sulfonylureas. This is caused by the additional glucose further stimulating insulin release in patients with intact pancreatic function (e.g., nondiabetics or patient with type-2 noninsulin dependent diabetes) [33, 34]. Given the similarities in biological mechanism to sulfonylureas, it is reasonable to propose that this phenomenon of rebound hypoglycemia may occur with fluoroquinolones as well. Although there are no specific therapies to reverse fluoroquinolone-induced hypoglycemia, octreotide may represent a novel adjunctive treatment given its well-documented success in sulfonylurea toxicity [35, 36].

Octreotide is a potent and long-acting synthetic analog of the inhibitory peptide hormone somatostatin [33]. Voltage-gated calcium channels on the pancreatic *β*-cell membrane are coupled to G-protein somatostatin-2 receptors. When these receptors are bound by octreotide, the voltage-gated calcium channels remain closed preventing calcium influx into the cell, therefore, inhibiting insulin release. This mechanism occurs downstream of the KATP channel thus inhibiting the cascade of molecular signaling induced by sulfonylureas and fluoroquinolones. Following the initial administration of octreotide to our patient, there was a significant increase in the ability to maintain euglycemia within 3 hours which was previously unable to be achieved with other supportive care therapies. Octreotide therapy also assisted in limiting the amount of infused fluids as the D10 infusion was discontinued 5.5 hours after the second dose. This benefit is important to consider when caring for patients with cardiac or renal failure where fluid overload can further complicate care.

The dosage and interval of octreotide therapy were based on published literature for insulin and sulfonylurea overdoses. In adults, doses of 50-100 mcg subcutaneously or intravenously every 6 to 12 hours have been reported [35]. Octreotide with its relatively benign adverse effect profile is well tolerated by most adult and pediatric patients [34, 36, 37]. The most common side effects are pain at the injection site and gastrointestinal disturbances. Given the anticipated short-term use of octreotide, the risks of this off-label therapy are minimal.

To our knowledge, this is the second case report to describe the successful use of octreotide for treating life-threatening hypoglycemia caused by levofloxacin in combination with glipizide. The first case by Kelesidis and Canseco was similar to ours in that an elderly woman with type-2 diabetes mellitus treated with glipizide and chronic kidney disease experienced refractory hypoglycemia to supportive measures complicated by seizures after one dose of ciprofloxacin [6]. After the administration of a single dose of 50 mcg octreotide intravenously, the patient's glucose normalized in approximately 2 hours. The time course for the ability to maintain euglycemia after the first subcutaneous octreotide administration was similar in our case at 3 hours. The difference in time to therapeutic effect could be explained by the different routes of administration. Conversely, the time to hypoglycemic event took 4 days to develop in our case in comparison to after a single dose. This highlights the importance of frequent monitoring for this adverse effect early and often after fluoroquinolone initiation.

This case is limited by the fact that causation cannot unequivocally establish levofloxacin as the sole cause of hypoglycemia given other potential contributing factors, including the use of sulfonylureas where both pharmacodynamic and pharmacokinetic mechanisms may have contributed. Based on the Naranjo score of 4, it is possible the hypoglycemia was attributed to levofloxacin [38]. Though appropriately dosed at the time of initiation days prior, our patient presented with acute renal insufficiency as evidenced by a greater than twofold increase in serum creatinine. This likely caused an accumulation of levofloxacin secondary to delayed renal clearance and a more pronounced dose-dependent pharmacodynamic effect. While all sulfonylureas independently increase the risk for hypoglycemia, it is unlikely that glipizide was the predominant driver of this adverse effect in this case. The patient had been on a stable dose of immediate release glipizide 10 mg daily for several years and there was no reason to suspect intentional overdose given that the patient came from a nursing rehabilitation facility. Moreover, glipizide is metabolized extensively by the liver yielding inactive metabolites with data to support its use in patients with chronic kidney disease or on dialysis. Given that our patient presented with normal LFTs, there is low suspicion there was a metabolism deficiency. Furthermore, the clearance of levofloxacin is three times longer than that of glipizide in patients with acute renal impairment [29, 30] Additionally, this patient had chronic low albumin suggesting the free drug fraction of glipizide has been steadily maintained and is an unlikely explanation for the acute effect seen in this case.

It should also be noted that the patient was initiated on high calorie, high fiber enteral nutrition (EN) at 50 mL/hr at the time octreotide was initiated. However, the carbohydrates provided by EN in addition to that provided by the D10 infusion at 100 mL/hr only supplied the patient with 20.8 g of carbohydrates per hour. This amount of carbohydrates is less than that provided by each bolus of D50 (25 g) and thus, unlikely to account for the sudden maintenance of euglycemia.

Our case and review of the literature highlight the safety concern of potential hypoglycemia with the use of levofloxacin in patients with identified risk factors and support the use of octreotide to reverse this phenomenon. Early recognition of this potential complication and subsequent treatment are necessary to prevent increased morbidity and mortality. Clinicians should exercise caution when prescribing fluoroquinolones by evaluating patients for known risk factors for hypoglycemia especially when unavoidable drug-drug interactions are possible. In patients unresponsive to standard supportive care therapies, the early adjunctive use of octreotide appears to be a reasonable off-label treatment option for fluoroquinolone-induced hypoglycemia.

## 4. Conclusion

Severe hypoglycemia is a known adverse effect of fluoroquinolones. However, until the recent updates in safety labeling, general awareness of this adverse effect was lacking. Clinicians should be aware of the risk factors and increase monitoring or choose alternative therapy when appropriate. It is essential clinicians are cognizant of these lesser-known adverse effects and drug-drug interactions to ensure prompt recognition and treatment using appropriate adjuncts. Octreotide may represent a novel reversal agent in addition to supportive care therapies for patients presenting with refractory, fluoroquinolone-induced hypoglycemia. To the best of the authors' knowledge, this is the second case report to describe the successful use of octreotide to treat severe and refractory hypoglycemia caused by levofloxacin in a patient with type-2 diabetes mellitus receiving glipizide.

## References

[1] Bolon M. K. (2011). The newer fluoroquinolones. *Medical Clinics of North America*.

[2] Safety alerts for human medical products - fluoroquinolone antibiotics: FDA requires labeling changes due to low blood sugar levels and mental health side effects. https://www.fda.gov/Safety/MedWatch/SafetyInformation/SafetyAlertsforHumanMedicalProducts/ucm612979.htm.

[3] Fusco S., Reitano F., Gambadoro N. (2013). Severe hypoglycemia associated with levofloxacin in a healthy older woman. *Journal of the American Geriatrics Society*.

[4] Bansal N., Manocha D., Madhira B. (2015). Life-threatening metabolic coma caused by levofloxacin. *American Journal of Therapeutics*.

[5] Parra-Riffo H., Lemus-Peñaloza J. (2012). Severe levofloxacin-induced hypoglycaemia: a case report and literature review. *Nefrologia*.

[6] Kelesidis T., Canseco E. (2010). Quinolone-induced hypoglycemia: a life-threatening but potentially reversible side effect. *American Journal of Medicine*.

[7] Kelesidis T., Canseco E. (2009). Levofloxacin-induced hypoglycemia: a rare but life-threatening side effect of a widely used antibiotic. *American Journal of Medicine*.

[8] Lawrence K. R., Adra M., Keir C. (2006). Hypoglycemia-induced anoxic brain injury possibly associated with levofloxacin. *Infection*.

[9] Friedrich L. V., Dougherty R. (2004). Fatal hypoglycemia associated with levofloxacin. *Pharmacotherapy*.

[10] Micheli L., Sbrilli M., Nencini C. (2012). Severe hypoglycemia associated with levofloxacin in Type 2 diabetic patients receiving polytherapy: two case reports. *International Journal of Clinical Pharmacology and Therapeutics*.

[11] Gibert A. E., Porta F. S. (2008). Hypoglycemia and levofloxacin: a case report. *Clinical Infectious Diseases*.

[12] Garber S. M., Pound M. W., Miller S. M. (2009). Hypoglycemia associated with the use of levofloxacin. *American Journal of Health-System Pharmacy*.

[13] Kanbay M., Aydogan T., Bozalan R. (2006). A rare but serious side effect of levofloxacin: hypoglycemia in a geriatric patient. *Diabetes Care*.

[14] FDA Drug Safety Communication https://www.fda.gov/downloads/Drugs/DrugSafety/UCM612834.pdf.

[15] Morales J., Schneider D. (2014). Hypoglycemia. *American Journal of Medicine*.

[16] Parekh T. M., Raji M., Lin Y.-L., Tan A., Kuo Y.-F., Goodwin J. S. (2014). Hypoglycemia after antimicrobial drug prescription for older patients using sulfonylureas. *JAMA Internal Medicine*.

[17] LaPlante K. L., Mersfelder T. L., Ward K. E., Quilliam B. J. (2008). Prevalence of and risk factors for dysglycemia in patients receiving gatifloxacin and levofloxacin in an outpatient setting. *Pharmacotherapy*.

[18] Chou H.-W., Wang J.-L., Chang C.-H., Lee J.-J., Shau W.-Y., Lai M.-S. (2013). Risk of severe dysglycemia among diabetic patients receiving levofloxacin, ciprofloxacin, or moxifloxacin in Taiwan. *Clinical Infectious Diseases*.

[19] Aspinall S. L., Good C. B., Jiang R., McCarren M., Dong D., Cunningham F. E. (2009). Severe dysglycemia with the fluoroquinolones: a class effect?. *Clinical Infectious Diseases*.

[20] Owens R. C., Ambrose P. G. (2005). Antimicrobial safety: focus on fluoroquinolones. *Clinical Infectious Diseases*.

[21] Koster J. C., Permutt M. A., Nichols C. G. (2005). Diabetes and insulin secretion: the ATP-sensitive K+ channel (K ATP) connection. *Diabetes*.

[22] Gloyn A. L., Pearson E. R., Antcliff J. F. (2004). Activating mutations in the gene encoding the ATP-sensitive potassium-channel subunit Kir6.2 and permanent neonatal diabetes. *The New England Journal of Medicine*.

[23] Maeda N., Tamagawa T., Niki I. (1996). Increase in insulin release from rat pancreatic islets by quinolone antibiotics. *British Journal of Pharmacology*.

[24] Saraya A., Yokokura M., Gonoi T., Seino S. (2004). Effects of fluoroquinolones on insulin secretion and beta-cell ATP-sensitive K+ channels. *European Journal of Pharmacology*.

[25] Sola D., Rossi L., Schianca G. P. C., Maffioli P., Bigliocca M., Mella R. (2015). Sulfonylureas and their use in clinical practice. *Archives of Medical Science*.

[26] Ghaly H., Kriete C., Sahin S. (2009). The insulinotropic effect of fluoroquinolones. *Biochemical Pharmacology*.

[27] Van Booven D., Marsh S., McLeod H. (2010). Cytochrome P450 2C9-CYP2C9. *Pharmacogenetics and Genomics*.

[28] Zhang L., Wei M., Zhao C., Qi H. (2008). Determination of the inhibitory potential of 6 fluoroquinolones on CYP1A2 and CYP2C9 in human liver microsomes. *Acta Pharmacologica Sinica*.

[29] Levofloxacin, Lexi-Drugs, Lexicomp [Internet]. http://online.lexi.com.

[30] Glipizide, Lexi-Drugs, Lexicomp [Internet]. http://online.lexi.com.

[31] Seedher N., Agarwal P. (2013). Competitive binding of fluoroquinolone antibiotics and some other drugs to human serum albumin: a luminescence spectroscopic study. *Luminescence*.

[32] Seedher N., Kanojia M. (2009). Mechanism of interaction of hypoglycemic agents glimepiride and glipizide with human serum albumin. *Central European Journal of Chemistry*.

[33] Lheureux P. E. R., Zahir S., Penaloza A., Gris M. (2005). Bench-to-bedside review: antidotal treatment of sulfonylurea-induced hypoglycaemia with octreotide. *Critical Care*.

[34] Dougherty P. P., Klein-Schwartz W. (2010). Octreotide’s role in the management of sulfonylurea-induced hypoglycemia. *Journal of Medical Toxicology*.

[35] Klein-Schwartz W., Stassinos G. L., Isbister G. K. (2016). Treatment of sulfonylurea and insulin overdose. *British Journal of Clinical Pharmacology*.

[36] Glatstein M., Scolnik D., Bentur Y. (2012). Octreotide for the treatment of sulfonylurea poisoning. *Clinical Toxicology*.

[37] Fasano C. J., O’Malley G., Dominici P., Aguilera E., Latta D. R. (2008). Comparison of octreotide and standard therapy versus standard therapy alone for the treatment of sulfonylurea-induced hypoglycemia. *Annals of Emergency Medicine*.

[38] Naranjo C. A., Busto U., Sellers E. M. (1981). A method for estimating the probability of adverse drug reactions. *Clinical Pharmacology & Therapeutics*.

